# Commercial Nucleic-Acid Amplification Tests for Diagnosis of Pulmonary Tuberculosis in Respiratory Specimens: Meta-Analysis and Meta-Regression

**DOI:** 10.1371/journal.pone.0001536

**Published:** 2008-02-06

**Authors:** Daphne I. Ling, Laura L. Flores, Lee W. Riley, Madhukar Pai

**Affiliations:** 1 Division of Epidemiology, School of Public Health, University of California, Berkeley, California, United States of America; 2 Division of Pulmonary and Critical Care Medicine, San Francisco General Hospital, San Francisco, California, United States of America; 3 Division of Infectious Diseases, School of Public Health, University of California, Berkeley, California, United States of America; 4 Department of Epidemiology, Biostatistics and Occupational Health, McGill University, Montreal, Quebec, Canada; National AIDS Research Institute, India

## Abstract

**Background:**

Hundreds of studies have evaluated the diagnostic accuracy of nucleic-acid amplification tests (NAATs) for tuberculosis (TB). Commercial tests have been shown to give more consistent results than in-house assays. Previous meta-analyses have found high specificity but low and highly variable estimates of sensitivity. However, reasons for variability in study results have not been adequately explored. We performed a meta-analysis on the accuracy of commercial NAATs to diagnose pulmonary TB and meta-regression to identify factors that are associated with higher accuracy.

**Methodology/Principal Findings:**

We identified 2948 citations from searching the literature. We found 402 articles that met our eligibility criteria. In the final analysis, 125 separate studies from 105 articles that reported NAAT results from respiratory specimens were included. The pooled sensitivity was 0.85 (range 0.36–1.00) and the pooled specificity was 0.97 (range 0.54–1.00). However, both measures were significantly heterogeneous (p<.001). We performed subgroup and meta-regression analyses to identify sources of heterogeneity. Even after stratifying by type of commercial test, we could not account for the variability. In the meta-regression, the threshold effect was significant (p = .01) and the use of other respiratory specimens besides sputum was associated with higher accuracy.

**Conclusions/Significance:**

The sensitivity and specificity estimates for commercial NAATs in respiratory specimens were highly variable, with sensitivity lower and more inconsistent than specificity. Thus, summary measures of diagnostic accuracy are not clinically meaningful. The use of different cut-off values and the use of specimens other than sputum could explain some of the observed heterogeneity. Based on these observations, commercial NAATs alone cannot be recommended to replace conventional tests for diagnosing pulmonary TB. Improvements in diagnostic accuracy, particularly sensitivity, need to be made in order for this expensive technology to be worthwhile and beneficial in low-resource countries.

## Introduction

Tuberculosis (TB) is a major global health problem. Each year, 8 to 9 million people develop disease, and 2 million die [1]. Pulmonary TB is the most common form of the disease [2]. Diagnosis of TB relies on the detection of acid-fast bacilli by microscopy (smear) and culture. Microscopy is rapid, specific, and inexpensive but has low sensitivity [3], [4]. Culture is more sensitive, but results can take several weeks. In addition, culture may be falsely-negative in 10–20% of cases [5]. Better efforts to control TB require faster and more accurate diagnostic tests [6]–[8]. Nucleic acid amplification tests (NAATs), which can give results in 3–6 hours, have been developed to address these issues [9].

The polymerase chain reaction (PCR) is the most common NAAT. Tests include those that are “in-house”, when they are based on a protocol developed in a non-commercial laboratory (“home-brew”), or commercial kits. Several commercial NAATs exist, and each uses a different method to amplify specific nucleic-acid regions in the *Mycobacterium tuberculosis* complex. These kits include: the GenProbe Amplified *M. tuberculosis* Direct test (AMTD), the Roche Amplicor MTB test, the Cobas Amplicor test, the Abbott LCx test, and the BD-ProbeTec (SDA) test. Another NAAT has been recently developed—the Loop-mediated Isothermal Amplification (LAMP) test, but research experience is limited with this test [10]. Table 1 provides a summary of the different commercial tests. The LCx kit is no longer in use, and Becton Dickinson has produced an enhanced version of the SDA test (BD-ProbeTec-ET). The Food and Drug Administration (FDA) has approved the use of select commercial NAATs for only respiratory specimens. In addition, the AMTD and Amplicor tests are licensed for testing smear-positive specimens, while the FDA recently approved a 2^nd^-generation AMTD (E-AMTD) test for smear-negative specimens [11]. The LCx, BD-ProbeTec-ET, and LAMP tests are currently not FDA-approved.

**Table 1:** Summary of Commercial Nucleic-Acid Amplification Tests (NAAT) for TB

NAAT	Manufacturer	Method
Amplified *M. tuberculosis* Direct Test (AMTD)	Gen-Probe Inc. San Diego, CA	Transcription-mediated amplification of rRNA
Amplicor MTB	Roche Molecular Systems Branchburg, NJ	PCR amplification of 16s rRNA
Cobas Amplicor	Roche Diagnostic Systems Mannheim, GERMANY	PCR amplification of 16s rRNA
LCx (discontinued)	Abbott Laboratories Abbott Park, IL	Ligase chain reaction amplication of 38kDa protein
BD-ProbeTec Direct (SDA)	Becton Dickinson Diagnostic Systems Sparks, MD	Strand displacement amplification of IS6110 and 16s rRNA
Loop-mediated Isothermal Amplification (LAMP)	Eiken Chemical Co. Ltd. Tokyo, JAPAN	Isothermal amplification and visual readout with UV fluorescence

Systematic reviews of previous studies have suggested that the diagnostic accuracy of NAATs varies more among in-house NAATs than commercial tests [12], [13]. A meta-analysis on the use of in-house PCR assays for testing sputum samples found significant heterogeneity and could not summarize the measures of diagnostic accuracy (i.e. sensitivity and specificity) [14]. Several meta-analyses have evaluated the accuracy of commercial NAATs in both pulmonary and extrapulmonary TB [12], [13], [15]–[17]. Most of them have reported high and consistent specificity but low and inconsistent estimates of sensitivity [12], [13], [15]. Smear-negative patients may be the most likely group to benefit from the use of NAATs. If the NAAT result is positive, a faster diagnosis can lead to an earlier initiation of therapy [11]. However, studies have shown that sensitivity is lower for smear-negative TB compared to smear-positive TB [12], [13], [15], [18]. One meta-analysis on the use of commercial NAATs for only smear-negative patients found that the sensitivity estimates were too low and variable to be used for confirming diagnosis in this group [16]. Another recent meta-analysis evaluated diagnostic accuracy for pulmonary TB stratified by smear status [18]. It concluded that the low sensitivity of smear-negative patients precludes the use of commercial NAATs for ruling out TB. Its high specificity in this group of patients, however, is useful for ruling in TB. The same study also noted that the high sensitivity in smear-positive samples could be helpful in ruling out a diagnosis of pulmonary TB due to infection by non-tuberculous mycobacteria (NTM) [18]. In our meta-analysis, we used a comprehensive search strategy to determine the accuracy of commercial NAATs for diagnosing pulmonary TB in combined smear-positive and smear-negative respiratory specimens. We further explore factors that may be accountable for differences among studies by meta-regression analysis.

## Methods

### Search strategy

We systematically searched the literature using predetermined inclusion criteria [19]. Criteria included: use of commercial NAATs on respiratory specimens for diagnosing pulmonary TB, comparison of NAAT result with culture as reference standard, information to calculate sensitivity and specificity, and minimum sample size of 50 to avoid selection bias [20]. We searched PUBMED (1985–2006), EMBASE (1988–2002), Web of Science (1990–2002), BIOSIS (1993–2002), Cochrane Library (2002; Issue 2), and LILACS (1990–2002). In addition, we reviewed the reference lists of several previously published reviews on NAATs [12]–[16], [18]. Further, we hand-searched the *Journal of Clinical Microbiology*, a high-yield journal for this review topic. Search terms included “tuberculosis, mycobacterium tuberculosis, nucleic acid amplification techniques, direct amplification test, polymerase chain reaction, ligase chain reaction, Amplicor, Cobas, Roche, Gen-Probe, Abbott, BD-ProbeTec, molecular diagnostic techniques, sensitivity and specificity, accuracy, and predictive value”. Reference lists from included studies were also searched. In addition, experts and commercial NAAT manufacturers were contacted for additional studies. This search criteria has been reported in previous meta-analyses [12]–[14].

### Study selection

**Figure 1:**
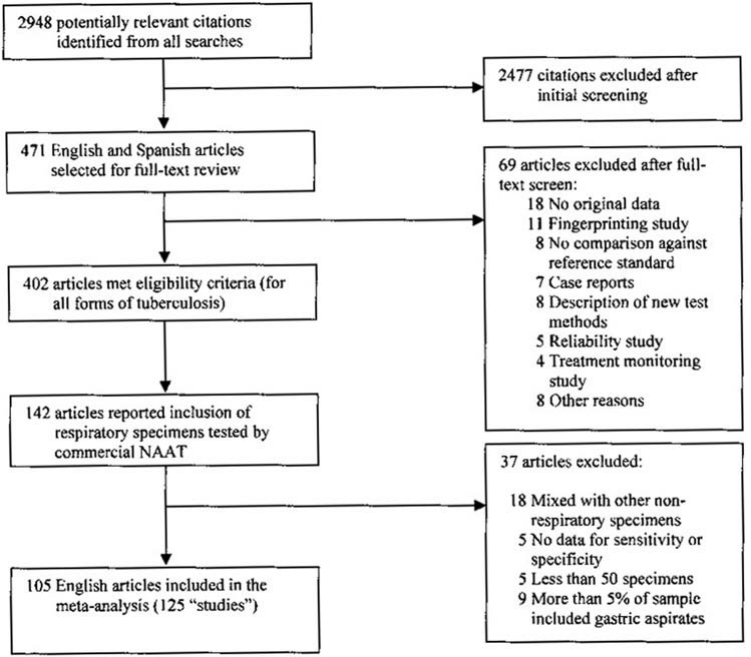
Study selection process.

We identified 2948 citations from the initial search. After screening titles and abstracts, 471 English and Spanish articles were eligible for full-text review. Of these, 69 articles were excluded, and 402 articles on the use of commercial NAATs for all forms of TB were included (screening done by two reviewers). A total of 142 articles focused on respiratory specimens [sputa, bronchial aspirates, bronchoalveolar lavages (BAL), and tracheal aspirates] for the diagnosis of pulmonary TB. Some articles considered gastric aspirates as respiratory specimens. They were accepted if the number of gastric aspirates was less than 5% of the total sample size. A total of 37 articles were further excluded from data extraction, and 105 articles were included in our meta-analysis [21]–[125]. Several articles compared more than one NAAT against the same reference standard in head-to-head trials, in which case each comparison was considered as a separate study. Thus, the total number of studies in the final analysis was 125. Figure 1 displays how the studies were selected.

### Data extraction

We created and piloted a data extraction form with a subset of eligible studies. Based upon experience gained in the pilot study, the data extraction form was finalized. The final set of studies was assessed with the standardized form by two reviewers (DIL and LLF), and any differences were resolved by consensus. Many articles compared NAAT results to more than one reference standard, and we used a hierarchical approach to choose one comparison from each study: (1) culture result plus clinical data (most preferred reference standard) (2) culture result alone and (3) clinical data alone (least preferred reference standard). We used the specimen as the unit of analysis when possible. We also chose to use data that were not subject to discrepant analyses (i.e. unresolved data) when available, since resolved data after discrepant analyses are a potential source of bias and result in higher estimates of accuracy [126]. In addition, NTM and inhibited specimens were excluded if possible.

### Assessment of study quality

We assessed the quality of studies using the following criteria, suggested as important for diagnostic studies [127]: (1) Was there a comparison of the commercial NAAT with an independent, appropriate reference standard? (2) Was the NAAT result interpreted without knowledge of the results of the reference standard (blinded interpretation) and vice-versa? (3) Did the whole sample or a randomly selected subset of the sample receive verification using the reference standard? and (4) Did the study prospectively recruit consecutive patients suspected of having pulmonary tuberculosis (i.e. cross-sectional vs case-control design)?

### Data synthesis and meta-analysis

Data were analyzed using Meta-Disc (version 1.4) software [128]. We pooled the data with the DerSimonian-Laird random effects model (REM) [20], [129]–[131]. The REM gives more conservative estimates with wider confidence intervals because it assumes that the meta-analysis includes only a sample of all possible studies [19], [132], [133]. In addition, the REM accounts for both within-study variability (random error) and between-study variability (heterogeneity). Accuracy measures include: sensitivity, specificity, positive likelihood ratio (LR+), negative likelihood ratio (LR-), and the diagnostic odds ratio (DOR). Sensitivity is the proportion of positive test results among those with the target disease. Specificity is the proportion of negative test results among those without the disease. In a clinical setting, likelihood ratios are considered useful. The LR+ measures how much more frequent a positive test is found in diseased versus non-diseased individuals. On the other hand, the LR- measures how more likely a negative result is found in diseased versus non-diseased individuals. The DOR, or the odds of a positive result in diseased individuals compared to the odds of a positive result in non-diseased individuals, combines both likelihood ratios and is a global measure of test performance [134]. A value of 1 would indicate that the test cannot discriminate between people with and without disease. The DOR is calculated by *LR+/LR−* or *[sensitivity/(1-specificity)]/[(1-sensitivity)/specificity]* [134].

Each study in the meta-analysis contributed a pair of numbers: sensitivity and specificity. Since these measures tend to be strongly correlated and vary with the thresholds (cut-off values for determining test positives) used across the individual studies, it is standard practice to analyze sensitivity and specificity proportions as pairs, and to also explore the effect of the threshold on study results. To do this, we performed the summary receiver operating characteristic (SROC) curve analysis [131], [135]. The SROC displays each study's sensitivity and specificity estimates within the ROC space. A regression curve is fitted through the distribution of pairs of sensitivity and specificity. A shoulder-like curve indicates that the variability between studies may be due to the threshold effect (i.e. variation in cut-off values used across studies) and that an underlying common DOR exists that does not change with the threshold [130], [135], [136]. A non shoulder-like curve shows that sensitivity and specificity are not correlated. The area under the regression curve also measures the overall accuracy of diagnostic tests. If the area under the curve (AUC) is 100%, then the test differentiates perfectly between diseased and non-diseased individuals. An AUC of 50% indicates poor diagnostic accuracy [130], [135], [136].

### Meta-regression

Heterogeneity in meta-analysis refers to a high degree of variability in study results (e.g. variability in sensitivity estimates). Such heterogeneity could be due to variability in thresholds (cut-off values), disease spectrum and populations studied, variations in NAAT protocols, and study quality across studies. When significant heterogeneity is present, summary estimates from meta-analyses are hard to interpret. We investigated heterogeneity using subgroup (stratified) analysis and meta-regression analysis [137]. In the subgroup analysis, we computed pooled DOR estimates in various strata to determine if accuracy is higher in specific subgroups.

The meta-regression analysis is an extension of the SROC model [135]. In this linear regression model, studies are the units of analysis. The DOR is the outcome (dependent) variable. The independent variables are the covariates that might be associated with the variability in the DOR. Based on previous meta-analyses [12]–[14], potentially relevant covariates for our meta-regression model included: prospective or retrospective study direction, recruitment method, blinded interpretation, type of test, specimen type, reference standard, and data resolution. There were insufficient numbers to compare categories of differing study design, degree of verification, and smear status.

The meta-regression model generates relative diagnostic odds ratios (RDOR) as the output [134], [137]. An RDOR is a ratio of two DORs. An RDOR of 1.0 indicates that a particular covariate (e.g. blinded study design) does not affect the overall DOR. An RDOR >1.0 indicates that studies with a particular characteristic (e.g. those that employed a specific target sequence in the PCR) have a higher DOR than studies without this characteristic. For a RDOR <1.0, the reverse holds.

## Results

The average sample size of the included studies was 715 (range 57–7539). With the exception of one study, all of our studies were cross-sectional. A majority (86%) of the studies were prospective in design. A total of 45 (36%) studies used consecutive or random sampling, while 29 (23%) studies recruited patients using some convenient sampling. The convenient sample was chosen from a bigger group of patients or was selected from a screening program. All but two studies reported complete verification of NAAT results with the same reference standard. Most of the studies (96%) collected both smear-positive and smear-negative specimens, and 84% compared NAAT results to culture as the reference standard. Ninety-five (76%) studies tested respiratory specimens, while 30 (24%) studies only used sputum specimens. We were able to analyze unresolved data (i.e. not subjected to discrepant analyses) in 88 (70%) studies. Past evidence has shown that investigators do not report all the study components in their publications [6], [138]. In our analysis, 103 (82%) studies did not report blinding status, and 51 (41%) studies did not explicitly report the method of patient recruitment. Table 2 gives the characteristics of the studies in our meta-analysis.

**Table 2:** Characteristics of NAAT Studies Included in the Review (N = 125)

Characteristic	Frequency (%)
**STUDY DIRECTION**	
Prospective	108 (86)
Retrospective	9 (7)
Both	8 (6)
**STUDY DESIGN**	
Cross-Sectional	124 (99)
**RECRUITMENT**	
Consecutive	43 (34)
Random	2 (2)
Convenient	24 (19)
Consecutive and Convenient	5 (4)
Not Reported	51 (41)
**VERIFICATION**	
Complete	123 (98)
**BLINDING**	
Both (double blind)	8 (6)
NAAT blinded to reference standard	7 (6)
Reference standard blinded to NAAT	5 (4)
None	2 (2)
Not Reported	103 (82)
**NAAT**	
Amplicor	34 (27)
Cobas Amplicor	18 (14)
AMTD	31 (25)
E-AMTD	9 (7)
LCx	18 (14)
BD-ProbeTec	6 (5)
BD-ProbeTec-ET	9 (7)
**SPECIMEN**	
Respiratory	95 (76)
Sputum	30 (24)
**REFERENCE STANDARD**	
Culture	105 (84)
Clinical Data	3 (2)
Culture and Clinical Data	17 (14)
**SMEAR STATUS**	
Both (positive and negative smears)	120 (96)
Negative	2 (2)
Not Reported	3 (2)
**DATA**	
Resolved (after discrepant analysis)	37 (30)
Not Resolved (discrepant analysis not done)	88 (70)

**Figure 2:**
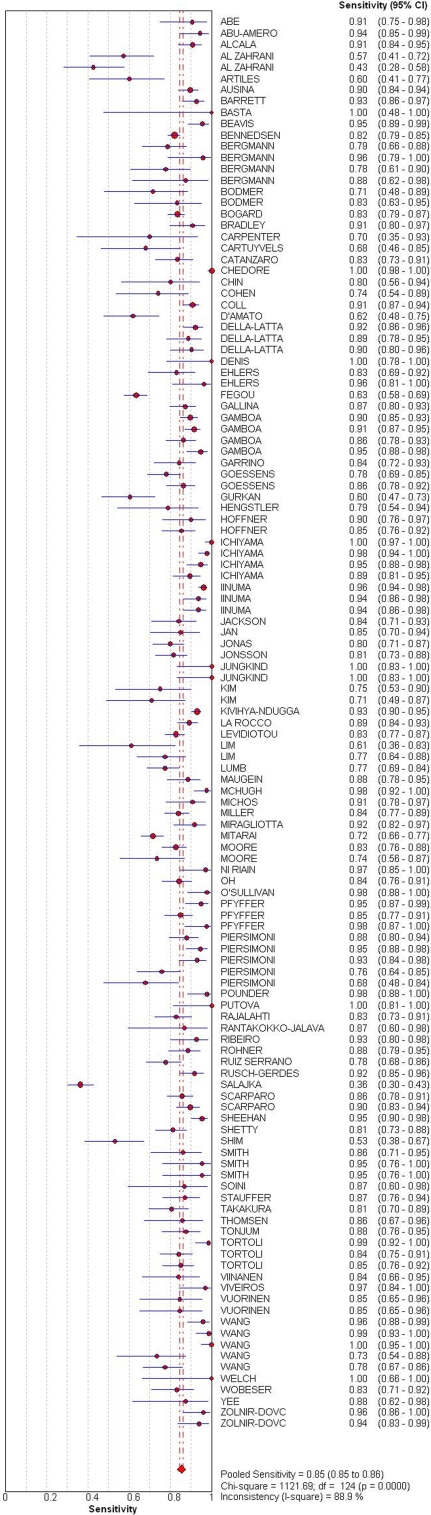
Forest plot of sensitivity estimates and 95% CI. Point estimates of sensitivity from each study are shown as solid circles. The solid lines represent the 95% confidence intervals (CI). Circles are proportional to study size. The pooled estimate is denoted by the diamond at the bottom.

**Figure 3:**
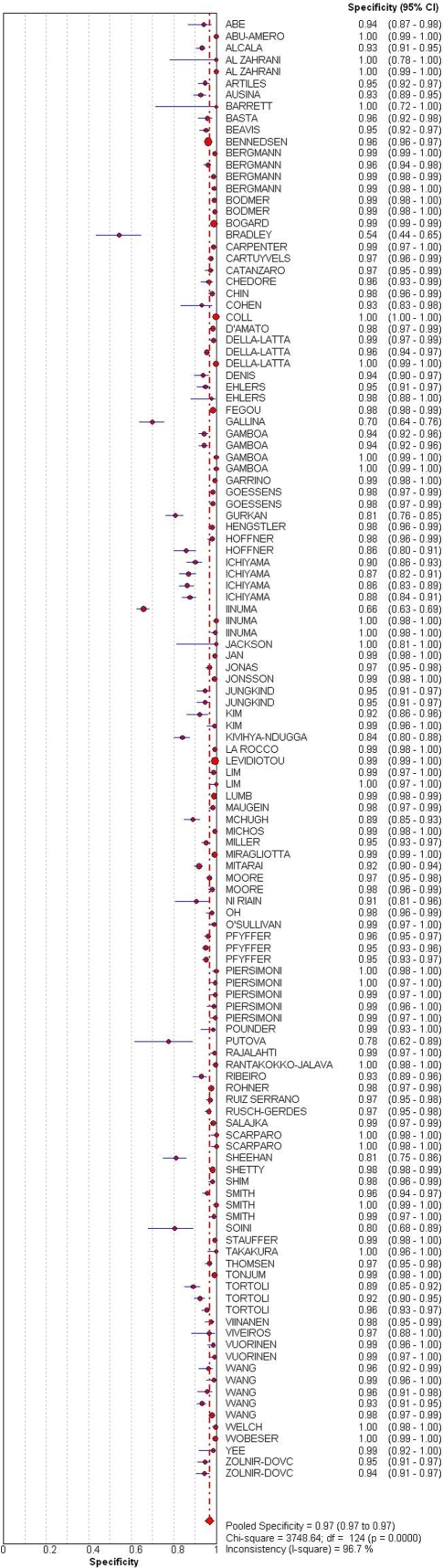
Forest plot of specificity estimates and 95% CI. Point estimates of specificity from each study are shown as solid circles. The solid lines represent the 95% confidence intervals (CI). Circles are proportional to study size. The pooled estimate is denoted by the diamond at the bottom.

The overall sensitivity and specificity estimates were 0.85 (range 0.36–1.00) and 0.97 (range 0.54–1.00), respectively. Figures 2 and 3 show the accuracy measures from all the studies in a forest plot. Specificity appears to be more consistent than sensitivity. Thirteen of 125 studies (10%) gave specificity estimates less than 90%. Most of them included either patients on treatment or who had history of prior disease. The overall LR+ was 32.74 (95% CI: 26.02, 41.22), and the overall LR- was 0.14 (95% CI: 0.12, 0.16). The pooled DOR was 268.88 (95% CI: 212.07, 340.9). We used Chi-square analysis to detect heterogeneity in the summary results. All of them showed highly significant heterogeneity (p<.001). Thus, pooled measures of the tests' diagnostic accuracy are not meaningful and do not adequately describe the data. Table 3 displays the accuracy measures and their corresponding statistics for the Chi-square test of heterogeneity.

**Table 3:** Pooled Summary Estimates of 125 Commercial NAAT Studies (adding 0.5 to all cells of studies with 0 values)

Accuracy Measure	Accuracy Estimate (95% Confidence Interval)	Chi^2^ test of heterogeneity	P value for heterogeneity
Sensitivity	0.85 (0.847, 0.86)	1121.69	<.001
Specificity	0.968 (0.967, 0.969)	3748.64	<.001
Positive Likelihood Ratio (LR+)	32.74 (26.01, 41.22)	3831.86	<.001
Negative Likelihood Ratio (LR-)	0.14 (0.12, 0.16)	1495.00	<.001
Diagnostic Odds Ratio (DOR)	268.88 (212.07, 340.9)	869.46	<.001

**Figure 4:**
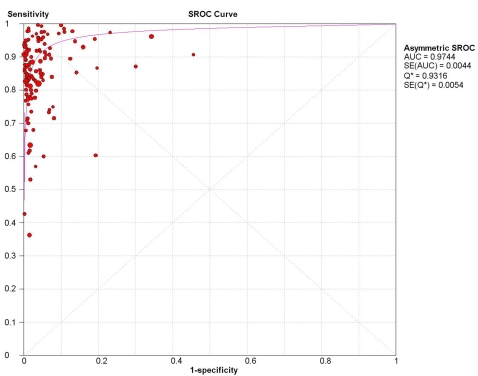
SROC plot with best-fitting asymmetric curve. Each solid circle represents each study in the meta-analysis. The curve is the regression line that summarizes the overall diagnostic accuracy. SROC = summary receiver operating characteristic; AUC = area under the curve; SE(AUC) = standard error of AUC; Q* = an index defined by the point on the SROC curve where the sensitivity and specificity are equal, which is the point closest to the top-left corner of the ROC space; SE(Q*) = standard error of Q* index.

Heterogeneity is a common concern for diagnostic meta-analyses. This variability may result from the threshold effect or differences in test methods and study characteristics [135]. Figure 4 shows the SROC plot with studies weighted by their inverse variance. The shoulder-like curve indicates that the threshold effect exists in our meta-analysis. There is a trade-off between sensitivity and specificity among the studies. Subgroup analysis is also used to identify other sources of variability by stratifying data into relatively more homogeneous strata [137]. Table 4 compares the DOR estimates for the study characteristics. The heterogeneity could be explained in some strata, but they consisted of small numbers. We stratified by type of commercial kit since they have standardized protocols. The variability in LR- did not persist for the SDA test (Table 5). The SDA test amplifies IS6110, which is usually present in high number of copies in MTB and may increase sensitivity. However, only 6 studies evaluated the SDA test, and significant heterogeneity remained for the other commercial NAATs.

**Table 4:** Diagnostic Odds Ratio (DOR) Estimates from Subgroup Analysis

Study Characteristic (n)	DOR	Chi^2^ test of heterogeneity	P value for heterogeneity
**DIRECTION**			
Prospective (108)	255.63 (199.23, 328.01)	678.67	<.001
Retrospective (9)	315.65 (99.68, 999.57)	150.21	<.001
Both (8)	371.42 (161.83, 852.49)	31.40	<.001
**STUDY DESIGN**			
Cross Sectional (124)	269.56 (212.30, 342.26)	869.08	<.001
**RECRUITMENT**			
Consecutive (43)	220.90 (154.41, 316.00)	180.24	<.001
Convenient (24)	347.98 (225.63, 536.67)	91.71	<.001
Both (5)	298.50 (90.72, 982.18)	40.54	<.001
Random (2)	278.72 (3.12, 24901.4)	9.73	0.002
Not Reported (51)	284.91 (184.02, 441.13)	529.38	<.001
**VERIFICATION**			
Complete (123)	264.79 (208.66, 336)	863.88	<.001
**BLINDING**			
Both (8)	163.93 (69.91, 384.42)	25.49	0.001
NAAT blinded (7)	446.86 (45.83, 4357.6)	106.41	<.001
Reference test blinded (5)	136.79 (76.13, 245.75)	4.55	0.337
Not Blinded (2)	84.26 (5.99, 1184.50)	5.39	0.020
Not Reported (103)	286.86 (223.72, 367.82)	681.83	<.001
**NAAT**			
Amplicor (34)	174.92 (120.77, 253.35)	198.52	<.001
Cobas Amplicor (18)	399.07 (238.32, 668.25)	83.93	<.001
AMTD (31)	298.05 (155.13, 572.62)	332.38	<.001
E-AMTD (9)	822.72 (194.22, 3485.1)	55.72	<.001
LCx (18)	215.60 (145.98, 318.44)	40.41	0.001
BD-ProbeTec (6)	424.45 (174.15, 1034.5)	10.96	0.052
BD-ProbeTec-ET (9)	266.86 (110.04, 647.19)	46.93	<.001
**SPECIMEN**			
Respiratory (95)	319.21 (247.88, 411.07)	546.49	<.001
Sputum (30)	138.91 (86.26, 223.70)	197.27	<.001
**REFERENCE STANDARD**			
Culture (105)	271.30 (211.67, 347.73)	688.15	<.001
Clinical Data (3)	70.30 (4.04, 1224.60)	40.06	<.001
Culture and Clinical (17)	300.84 (163.1, 554.92)	70.57	<.001
**SMEAR STATUS**			
Both (120)	270.79 (212.77, 344.63) 61.79 (17.83, 214.14)	837.09	<.001
Negative (2)	828.06 (317.8, 2157.6)	3.14	0.076
Not Reported (3)		0.04	0.982
**DATA**			
Resolved (37)	254.01 (177.34, 363.81)	200.87	<.001
Not Resolved (88)	278.33 (203.79, 380.13)	668.45	<.001

**Table 5:** Likelihood Ratios Stratified by Commerical NAAT

Test	Positive Likelihood Ratio (95% CI)	P value for heterogeneity	Negative Likelihood Ratio (95% CI)	P value for heterogeneity
Amplicor	26.04 (17.04, 39.80)	<.001	0.15 (0.11, 0.22)	<.001
Cobas Amplicor	58.59 (37.77, 90.86)	<.001	0.17 (0.13, 0.22)	<.001
AMTD	28.75 (17.79, 46.47)	<.001	0.12 (0.09, 0.17)	<.001
E-AMTD	57.55 (25.49, 129.92)	<.001	0.12 (0.07, 0.22)	<.001
LCx	26.91 (17.21, 42.09)	<.001	0.16 (0.12, 0.20)	<.001
BD-ProbeTec	20.11 (10.42, 38.82)	<.001	0.06 (0.04, 0.10)	0.264
BD-ProbeTec-ET	37.07 (19.18, 71.65)	<.001	0.14 (0.09, 0.20)	0.002

A meta-regression analysis was performed to help explain the variation even after subgroup analysis. Table 6 shows the RDOR estimates from the meta-regression analysis using the Restricted Maximum Likelihood (REML) method to measure between-study variance. The threshold effect (S) = −0.21 was significant (p = 0.01) in accordance with the SROC plot. The “S” coefficient is a way to measure the effect of different thresholds on the DOR among studies, and the negative value indicates that the thresholds increase specificity at the expense of sensitivity [16]. Thus, the heterogeneity found in our meta-analysis could be explained in part by the use of different cut-off values in the studies. In addition, studies that evaluated respiratory specimens had almost a two-fold increase in DOR compared to studies that used only sputum. None of the other covariates in the model reached statistical significance. Previous meta-analyses have shown that including bronchial specimens gave higher accuracy estimates compared to studies that only collected sputum [16], [18].

**Table 6:** Results from Meta-Regression Analysis Using the Restricted Maximum Likelihood Method

Comparison	Model Coefficient	Relative Diagnostic Odds Ratio (95% CI)	P value
Threshold Effect (S)	−0.21	—	0.01
Retrospective/Both (17) vs Prospective Design (108)	0.13	1.14 (0.56, 2.33)	0.71
Some Convenient Sampling/NR (80) vs Consecutive/Random Sampling (45)	0.38	1.46 (0.87, 2.43)	0.15
No Blinding/NR (105) vs Any Blinding (20)	0.25	1.29 (0.65, 2.58)	0.47
FDA-Approved NAATs (92) vs Not FDA-Approved NAATs (33)	−0.06	0.95 (0.53, 1.68)	0.85
Respiratory Specimens (95) vs Sputum Specimens (30)	0.64	1.89 (1.01, 3.52)	0.05
Culture Reference Standard (105) vs Clinical Reference/Both (20)	0.34	1.40 (0.70, 2.81)	0.34
Resolved Data (37) vs Unresolved Data (88)	−0.05	0.95 (0.54, 1.66)	0.86

## Discussion

### Principal findings

Lack of rapid and accurate diagnostics for TB has been a major concern for global TB control. NAATs were introduced as promising novel tests for TB, and several commercial assays were introduced into the market. However, their actual performance has been less than optimal [12]–[18]. Since hundreds of studies have been published on NAATs, there is now the opportunity to perform meta-analyses and meta-regression to explore factors that influence NAAT performance.

In this meta-analysis, we performed extensive literature searches and identified a total of 125 separate studies from 105 articles that reported NAAT results from respiratory specimens. The results showed that sensitivity and specificity estimates for commercial NAATs in respiratory specimens were highly variable, with sensitivity lower and more inconsistent than specificity. Thus, summary measures of diagnostic accuracy are not clinically meaningful. The use of different cut-off values and the use of specimens other than sputum could explain some of the observed heterogeneity.

### Implications of the findings

The most notable advantage of commercial NAATs is their rapid turn-around time, which may have important implications for patient management and TB control. However, they appear to be impacted by a trade-off between sensitivity and specificity—specificity appears maximized at the cost of sensitivity. Reasons to account for their low sensitivity include low concentration of bacilli (i.e. paucibacillary specimens), such as smear-negative sputum specimens, or the presence of inhibitory substances [139]. We did not find high rates of inhibition in the studies reviewed (range 1%–7.5%). In addition, the small volumes of specimen (template) used in each commercial test may offer additional explanations. A recent meta-analysis on NAATs for TB lymphadenitis found that studies which used volumes of template >20 µl were more accurate than studies that used lesser template volumes [17]. Furthermore, study results may be influenced by the reference standard used to compare test results. It is well known that culture is not 100% sensitive and can give false-negative results. The lack of a diagnostic gold standard remains one of the biggest obstacles for evaluating new diagnostics, especially in HIV-infected persons and in paucibacillary disease (e.g. extrapulmonary TB and pediatric disease). The true accuracy of commercial NAATs may actually be higher than reported when using an imperfect reference standard [140].

Our results show a high degree of variability in accuracy across studies. The increased power of a meta-analysis can determine a test's overall diagnostic ability, but a summary measure is misleading in the presence of significant heterogeneity. In previous meta-analyses [12]–[14], subgroup analyses did not fully explain the variability found in NAAT results across studies. Even when stratifying by commercial test, our results remained heterogeneous. Other setting-specific factors, such as background TB prevalence rates or laboratory experience, could help account for this variation. Aside from the threshold effect, meta-regression analysis found that studies which collected several types of respiratory specimens were associated with higher diagnostic accuracy, possibly since the induction of aspirates yields a higher recovery of bacteria. Our findings agree with previous meta-analyses that suggest commercial NAATs cannot replace culture and microscopy but should be interpreted along with conventional tests and clinical data for diagnosing TB [12], [13], [15]. NAATs are also not useful for monitoring treatment progress since they can detect non-viable bacteria and give false-positive results [141]. However, they can distinguish *M. tuberculosis* from NTM [9]. This may be helpful in high-NTM populations, such as HIV/AIDS patients.

### Limitations of NAAT studies

Systematic reviews and meta-analyses are critical for evidence-based clinical practice [131], [142]. However, they are only as good as the quality of the studies that they include. There is growing concern that primary research on TB diagnostics are not methodologically rigorous [143], [144]. In a review of 12 recent meta-analyses of various TB tests, studies were plagued by limitations such as lack of blinding, use of a case-control design, and lack of random or consecutive patient sampling methodology [6]. One review of 31 meta-analyses on several diseases found higher accuracy measures associated with studies that used non-consecutive sampling methods [138]. In our meta-regression, the use of some convenience sampling gave a DOR that was 1.5-fold higher than the DOR for studies that used random or consecutive sampling. This finding was almost significant (p = 0.15). In addition, 41% of our studies did not report how their patients were recruited. Thus, besides poor methodological quality, poor reporting of study components is another problem [6]. In our meta-analysis, 82% of the studies did not report blinding status. Not blinding investigators to reference standard results when interpreting the NAAT test has been shown to overestimate the DOR [13], [16], [145]. Another limitation of existing NAAT studies is lack of data on whether NAATs actually have an impact on patient outcomes and how much value NAATs contribute, over and above the information already obtained by conventional testing. Most studies only provided information on sensitivity and specificity.

### Strengths and limitations of the systematic review

Our systematic review had several strengths. First, we used a comprehensive search strategy with various overlapping approaches. This enabled us to retrieve a large number of studies. Moreover, two reviewers independently completed screening, study selection, and data extraction. Finally, we analyzed data within specific subgroups to lessen the effect of heterogeneity and used meta-regression to identify factors associated with higher accuracy. Our review had limitations as well. Despite searching several sources, it is possible that we may have missed some eligible studies. Further, we could only extract data from English language studies, and this could have introduced bias in our results. Lastly, despite using subgroup analysis and meta-regression methods, considerable heterogeneity remained unexplained.

Even if sensitivity were to be improved, an important issue that will remain is the implementation of these new tools in developing countries. Commercial kits, whose prices range from US$25–50 per test, are popular in the US and other developed countries [9], [11]. The US Center for Disease Control and Prevention (CDC) has reported that commercial NAATs are used mostly in hospitals, health departments, and independent laboratories in the US [146]. However, many developing countries still use in-house PCR assays, which only cost about $15 per test [147]. Ironically, the poorest countries are often the ones burdened by the highest number of cases and therefore unlikely to benefit from expensive technologies. Realizing this, agencies such as the Foundation for Innovative New Diagnostics (FIND), the WHO, and the Stop TB Working Group for New Diagnostics have launched initiatives to make technologies for detecting TB and other neglected diseases affordable and accessible for developing countries [148].

## References

[1] WHO (2006). Global Tuberculosis Control: Surveillance, Planning, Financing..

[2] Dye C, Scheele S, Dolin P, Pathania V, Raviglione MC (1999). Consensus statement. Global burden of tuberculosis: estimated incidence, prevalence, and mortality by country. WHO Global Surveillance and Monitoring Project.. Jama.

[3] Steingart KR, Henry M, Ng V, Hopewell PC, Ramsay A (2006). Fluorescence versus conventional sputum smear microscopy for tuberculosis: a systematic review.. Lancet Infect Dis.

[4] Steingart KR, Ng V, Henry M, Hopewell PC, Ramsay A (2006). Sputum processing methods to improve the sensitivity of smear microscopy for tuberculosis: a systematic review.. Lancet Infect Dis.

[5] Pai M (2004). The accuracy and reliability of nucleic acid amplification tests in the diagnosis of tuberculosis.. Natl Med J India.

[6] Pai M, O'Brien R (2006). Tuberculosis diagnostics trials: do they lack methodological rigor?. Expert Rev Mol Diagn.

[7] Pai M, Kalantri S, Dheda K (2006). New tools and emerging technologies for the diagnosis of tuberculosis: part II. Active tuberculosis and drug resistance.. Expert Rev Mol Diagn.

[8] Pai M, Kalantri S, Dheda K (2006). New tools and emerging technologies for the diagnosis of tuberculosis: part I. Latent tuberculosis.. Expert Rev Mol Diagn.

[9] (1997). Rapid diagnostic tests for tuberculosis: what is the appropriate use? American Thoracic Society Workshop.. Am J Respir Crit Care Med.

[10] Boehme CC, Nabeta P, Henostroza G, Raqib R, Rahim Z (2007). Operational feasibility of using loop-mediated isothermal amplification for diagnosis of pulmonary tuberculosis in microscopy centers of developing countries.. J Clin Microbiol.

[11] (2000). Update: Nucleic acid amplification tests for tuberculosis.. MMWR Morb Mortal Wkly Rep.

[12] Pai M, Flores LL, Hubbard A, Riley LW, Colford JM (2004). Nucleic acid amplification tests in the diagnosis of tuberculous pleuritis: a systematic review and meta-analysis.. BMC Infect Dis.

[13] Pai M, Flores LL, Pai N, Hubbard A, Riley LW (2003). Diagnostic accuracy of nucleic acid amplification tests for tuberculous meningitis: a systematic review and meta-analysis.. Lancet Infect Dis.

[14] Flores LL, Pai M, Colford JM, Riley LW (2005). In-house nucleic acid amplification tests for the detection of Mycobacterium tuberculosis in sputum specimens: meta-analysis and meta-regression.. BMC Microbiol.

[15] Piersimoni C, Scarparo C (2003). Relevance of commercial amplification methods for direct detection of Mycobacterium tuberculosis complex in clinical samples.. J Clin Microbiol.

[16] Sarmiento OL, Weigle KA, Alexander J, Weber DJ, Miller WC (2003). Assessment by meta-analysis of PCR for diagnosis of smear-negative pulmonary tuberculosis.. J Clin Microbiol.

[17] Daley PTS, Pai M (2007). Nucleic acid amplification tests for the diagnosis of tuberculous lymphadenitis: a systematic review.. Int J Tuberc Lung Dis.

[18] Greco S, Girardi E, Navarra A, Saltini C (2006). Current evidence on diagnostic accuracy of commercially based nucleic acid amplification tests for the diagnosis of pulmonary tuberculosis.. Thorax.

[19] Egger MSG, Altman DG (2001). Systematic reviews in health care. Meta-analysis in context..

[20] Deville WL, Buntinx F, Bouter LM, Montori VM, de Vet HC (2002). Conducting systematic reviews of diagnostic studies: didactic guidelines.. BMC Med Res Methodol.

[21] Abe C, Hirano K, Wada M, Kazumi Y, Takahashi M (1993). Detection of Mycobacterium tuberculosis in clinical specimens by polymerase chain reaction and Gen-Probe Amplified Mycobacterium Tuberculosis Direct Test.. J Clin Microbiol.

[22] Abu-Amero KK (2002). Potential for the Use of Polymerase Chain Reaction (PCR) in the Detection and Identification of Mycobacterium tuberculosis Complex in Sputum Samples.. Molecular Biology Today.

[23] Alcala L, Ruiz-Serrano MJ, Hernangomez S, Marin M, Garcia de Viedma D (2001). Evaluation of the upgraded amplified Mycobacterium tuberculosis direct test (gen-probe) for direct detection of Mycobacterium tuberculosis in respiratory and non-respiratory specimens.. Diagn Microbiol Infect Dis.

[24] Al Zahrani K, Al Jahdali H, Poirier L, Rene P, Menzies D (2001). Yield of smear, culture and amplification tests from repeated sputum induction for the diagnosis of pulmonary tuberculosis.. Int J Tuberc Lung Dis.

[25] Artiles F, Jose Pena M, Isolina Campos-Herrero M, Lafarga B (2001). [Clinical evaluation of the Amplified Mycobacterium Tuberculosis Direct 2 test].. Enferm Infecc Microbiol Clin.

[26] Ausina V, Gamboa F, Gazapo E, Manterola JM, Lonca J (1997). Evaluation of the semiautomated Abbott LCx Mycobacterium tuberculosis assay for direct detection of Mycobacterium tuberculosis in respiratory specimens.. J Clin Microbiol.

[27] Barrett A, Magee JG, Freeman R (2002). An evaluation of the BD ProbeTec ET system for the direct detection of Mycobacterium tuberculosis in respiratory samples.. J Med Microbiol.

[28] Basta PC, Oelemann MA, Oelemann WM, Fonseca Lde S, Coimbra CE (2006). Detection of Mycobacterium tuberculosis in sputum from Surui Indian subjects, Brazilian Amazon.. Mem Inst Oswaldo Cruz.

[29] Beavis KG, Lichty MB, Jungkind DL, Giger O (1995). Evaluation of Amplicor PCR for direct detection of Mycobacterium tuberculosis from sputum specimens.. J Clin Microbiol.

[30] Bennedsen J, Thomsen VO, Pfyffer GE, Funke G, Feldmann K (1996). Utility of PCR in diagnosing pulmonary tuberculosis.. J Clin Microbiol.

[31] Bergmann JS, Woods GL (1996). Clinical evaluation of the Roche AMPLICOR PCR Mycobacterium tuberculosis test for detection of M. tuberculosis in respiratory specimens.. J Clin Microbiol.

[32] Bergmann JS, Woods GL (1998). Clinical evaluation of the BDProbeTec strand displacement amplification assay for rapid diagnosis of tuberculosis.. J Clin Microbiol.

[33] Bergmann JS, Yuoh G, Fish G, Woods GL (1999). Clinical evaluation of the enhanced Gen-Probe Amplified Mycobacterium Tuberculosis Direct Test for rapid diagnosis of tuberculosis in prison inmates.. J Clin Microbiol.

[34] Bergmann JS, Keating WE, Woods GL (2000). Clinical evaluation of the BDProbeTec ET system for rapid detection of Mycobacterium tuberculosis.. J Clin Microbiol.

[35] Bodmer T, Gurtner A, Schopfer K, Matter L (1994). Screening of respiratory tract specimens for the presence of Mycobacterium tuberculosis by using the Gen-Probe Amplified Mycobacterium Tuberculosis Direct Test.. J Clin Microbiol.

[36] Bodmer T, Mockl E, Muhlemann K, Matter L (1996). Improved performance of Gen-Probe Amplified Mycobacterium Tuberculosis Direct Test when 500 instead of 50 microliters of decontaminated sediment is used.. J Clin Microbiol.

[37] Bogard M, Vincelette J, Antinozzi R, Alonso R, Fenner T (2001). Multicenter study of a commercial, automated polymerase chain reaction system for the rapid detection of Mycobacterium tuberculosis in respiratory specimens in routine clinical practice.. Eur J Clin Microbiol Infect Dis.

[38] Bradley SP, Reed SL, Catanzaro A (1996). Clinical efficacy of the amplified Mycobacterium tuberculosis direct test for the diagnosis of pulmonary tuberculosis.. Am J Respir Crit Care Med.

[39] Carpentier E, Drouillard B, Dailloux M, Moinard D, Vallee E (1995). Diagnosis of tuberculosis by Amplicor Mycobacterium tuberculosis test: a multicenter study.. J Clin Microbiol.

[40] Cartuyvels R, De Ridder C, Jonckheere S, Verbist L, Van Eldere J (1996). Prospective clinical evaluation of Amplicor Mycobacterium tuberculosis PCR test as a screening method in a low-prevalence population.. J Clin Microbiol.

[41] Catanzaro A, Perry S, Clarridge JE, Dunbar S, Goodnight-White S (2000). The role of clinical suspicion in evaluating a new diagnostic test for active tuberculosis: results of a multicenter prospective trial.. Jama.

[42] Chedore P, Jamieson FB (1999). Routine use of the Gen-Probe MTD2 amplification test for detection of Mycobacterium tuberculosis in clinical specimens in a large public health mycobacteriology laboratory.. Diagn Microbiol Infect Dis.

[43] Chin DP, Yajko DM, Hadley WK, Sanders CA, Nassos PS (1995). Clinical utility of a commercial test based on the polymerase chain reaction for detecting Mycobacterium tuberculosis in respiratory specimens.. Am J Respir Crit Care Med.

[44] Cohen RA, Muzaffar S, Schwartz D, Bashir S, Luke S (1998). Diagnosis of pulmonary tuberculosis using PCR assays on sputum collected within 24 hours of hospital admission.. Am J Respir Crit Care Med.

[45] Coll P, Garrigo M, Moreno C, Marti N (2003). Routine use of Gen-Probe Amplified Mycobacterium Tuberculosis Direct (MTD) test for detection of Mycobacterium tuberculosis with smear-positive and smear-negative specimens.. Int J Tuberc Lung Dis.

[46] D'Amato RF, Wallman AA, Hochstein LH, Colaninno PM, Scardamaglia M (1995). Rapid diagnosis of pulmonary tuberculosis by using Roche AMPLICOR Mycobacterium tuberculosis PCR test.. J Clin Microbiol.

[47] Della-Latta P, Whittier S (1998). Comprehensive evaluation of performance, laboratory application, and clinical usefulness of two direct amplification technologies for the detection of Mycobacterium tuberculosis complex.. Am J Clin Pathol.

[48] Denis O, Devaster JM, Vandenberg O, Vanachter H, Lafontaine T (1998). Evaluation of ligase chain reaction for direct detection of Mycobacterium tuberculosis in respiratory specimens.. Zentralbl Bakteriol.

[49] Ehlers S, Pirmann M, Zaki W, Hahn H (1994). Evaluation of a commercial rRNA target amplification assay for detection of Mycobacterium tuberculosis complex in respiratory specimens.. Eur J Clin Microbiol Infect Dis.

[50] Ehlers S, Ignatius R, Regnath T, Hahn H (1996). Diagnosis of extrapulmonary tuberculosis by Gen-Probe amplified Mycobacterium tuberculosis direct test.. J Clin Microbiol.

[51] Fegou E, Jelastopulu E, Sevdali M, Anastassiou ED, Dimitracopoulos G (2005). Sensitivity of the Cobas Amplicor system for detection of Mycobacterium tuberculosis in respiratory and extrapulmonary specimens.. Clin Microbiol Infect.

[52] Gallina M, Troupioti P, Rocco G, Sensalari G, Libanori E (2000). Predicting culture results for Mycobacterium tuberculosis complex. Amplified mycobacterium tuberculosis direct test and acid-fast bacilli microscopy.. Chest.

[53] Gamboa F, Fernandez G, Padilla E, Manterola JM, Lonca J (1998). Comparative evaluation of initial and new versions of the Gen-Probe Amplified Mycobacterium Tuberculosis Direct Test for direct detection of Mycobacterium tuberculosis in respiratory and nonrespiratory specimens.. J Clin Microbiol.

[54] Gamboa F, Manterola JM, Lonca J, Matas L, Cardona PJ (1998). Comparative evaluation of two commercial assays for direct detection of Mycobacterium tuberculosis in respiratory specimens.. Eur J Clin Microbiol Infect Dis.

[55] Garrino MG, Glupczynski Y, Degraux J, Nizet H, Delmee M (1999). Evaluation of the Abbott LCx Mycobacterium tuberculosis assay for direct detection of Mycobacterium tuberculosis complex in human samples.. J Clin Microbiol.

[56] Goessens WH, de Man P, Koeleman JG, Luijendijk A, te Witt R (2005). Comparison of the COBAS AMPLICOR MTB and BDProbeTec ET assays for detection of Mycobacterium tuberculosis in respiratory specimens.. J Clin Microbiol.

[57] Gurkan O, Acican T, Gulbay B (2002). Evaluation of an amplified Mycobacterium tuberculosis direct test in clinical specimens.. Int J Tuberc Lung Dis.

[58] Hengstler M KP, Glockner G, Fahr AM (1996). Evaluation of the Amplicor Mycobacterium tuberculosis amplification and detection kit in a clinical laboratory: results and experiences.. Clin Lab.

[59] Hoffner SE, Cristea M, Klintz L, Petrini B, Kallenius G (1996). RNA amplification for direct detection of mycobacterium tuberculosis in respiratory samples.. Scand J Infect Dis.

[60] Hoffner SE, Norberg R, Carlos Toro J, Winqvist N, Koivula T (1996). Direct detection of Mycobacterium tuberculosis in sputum samples from Guinea Bissau by an rRNA target-amplified test system.. Tuber Lung Dis.

[61] Ichiyama S, Iinuma Y, Tawada Y, Yamori S, Hasegawa Y (1996). Evaluation of Gen-Probe Amplified Mycobacterium Tuberculosis Direct Test and Roche PCR-microwell plate hybridization method (AMPLICOR MYCOBACTERIUM) for direct detection of mycobacteria.. J Clin Microbiol.

[62] Ichiyama S, Ito Y, Sugiura F, Iinuma Y, Yamori S (1997). Diagnostic value of the strand displacement amplification method compared to those of Roche Amplicor PCR and culture for detecting mycobacteria in sputum samples.. J Clin Microbiol.

[63] Iinuma Y, Ichiyama S, Yamori S, Oohama J, Takagi N (1998). Diagnostic value of the Amplicor PCR assay for initial diagnosis and assessment of treatment response for pulmonary tuberculosis.. Microbiol Immunol.

[64] Iinuma Y, Senda K, Fujihara N, Saito T, Takakura S (2003). Comparison of the BDProbeTec ET system with the Cobas Amplicor PCR for direct detection of Mycobacterium tuberculosis in respiratory samples.. Eur J Clin Microbiol Infect Dis.

[65] Jackson KM, Edwards RM, Bowden DS, Leslie DE (1996). Evaluation of the Roche Amplicor polymerase chain reaction system for detection of Mycobacterium tuberculosis complex in specimens.. Pathology.

[66] Jan IS, Hsueh PR, Teng LJ, Lee LN, Yang PC (1998). Evaluation of an automatic polymerase chain reaction assay for identification of Mycobacterium tuberculosis in respiratory specimens.. J Formos Med Assoc.

[67] Jonas V, Alden MJ, Curry JI, Kamisango K, Knott CA (1993). Detection and identification of Mycobacterium tuberculosis directly from sputum sediments by amplification of rRNA.. J Clin Microbiol.

[68] Jonsson B, Ridell M (2003). The Cobas Amplicor MTB test for detection of Mycobacterium tuberculosis complex from respiratory and non-respiratory clinical specimens.. Scand J Infect Dis.

[69] Jungkind D, Direnzo S, Beavis KG, Silverman NS (1996). Evaluation of automated COBAS AMPLICOR PCR system for detection of several infectious agents and its impact on laboratory management.. J Clin Microbiol.

[70] Kim SY, Park YJ, Kang SJ, Kim BK, Kang CS (2004). Comparison of the BDProbeTec ET system with the roche COBAS AMPLICOR System for detection of Mycobacterium tuberculosis complex in the respiratory and pleural fluid specimens.. Diagn Microbiol Infect Dis.

[71] Kivihya-Ndugga L, van Cleeff M, Juma E, Kimwomi J, Githui W (2004). Comparison of PCR with the routine procedure for diagnosis of tuberculosis in a population with high prevalences of tuberculosis and human immunodeficiency virus.. J Clin Microbiol.

[72] La Rocco MT, Wanger A, Ocera H, Macias E (1994). Evaluation of a commercial rRNA amplification assay for direct detection of Mycobacterium tuberculosis in processed sputum.. Eur J Clin Microbiol Infect Dis.

[73] Levidiotou S, Vrioni G, Galanakis E, Gesouli E, Pappa C (2003). Four-year experience of use of the Cobas Amplicor system for rapid detection of Mycobacterium tuberculosis complex in respiratory and nonrespiratory specimens in Greece.. Eur J Clin Microbiol Infect Dis.

[74] Lim TK, Gough A, Chin NK, Kumarasinghe G (2000). Relationship between estimated pretest probability and accuracy of automated Mycobacterium tuberculosis assay in smear-negative pulmonary tuberculosis.. Chest.

[75] Lim TK, Mukhopadhyay A, Gough A, Khoo KL, Khoo SM (2003). Role of clinical judgment in the application of a nucleic acid amplification test for the rapid diagnosis of pulmonary tuberculosis.. Chest.

[76] Lumb R, Davies K, Dawson D, Gibb R, Gottlieb T (1999). Multicenter evaluation of the Abbott LCx Mycobacterium tuberculosis ligase chain reaction assay.. J Clin Microbiol.

[77] Maugein J, Fourche J, Vacher S, Grimond C, Bebear C (2002). Evaluation of the BDProbeTec ET DTB assay(1) for direct detection of Mycobacterium tuberculosis complex from clinical samples.. Diagn Microbiol Infect Dis.

[78] McHugh TD, Pope CF, Ling CL, Patel S, Billington OJ (2004). Prospective evaluation of BDProbeTec strand displacement amplification (SDA) system for diagnosis of tuberculosis in non-respiratory and respiratory samples.. J Med Microbiol.

[79] Michos AG, Daikos GL, Tzanetou K, Theodoridou M, Moschovi M (2006). Detection of Mycobacterium tuberculosis DNA in respiratory and nonrespiratory specimens by the Amplicor MTB PCR.. Diagn Microbiol Infect Dis.

[80] Miller N, Hernandez SG, Cleary TJ (1994). Evaluation of Gen-Probe Amplified Mycobacterium Tuberculosis Direct Test and PCR for direct detection of Mycobacterium tuberculosis in clinical specimens.. J Clin Microbiol.

[81] Miragliotta G, Antonetti R, Di Taranto A, Mosca A, Del Prete R (2005). Direct detection of Mycobacterium tuberculosis complex in pulmonary and extrapulmonary samples by BDProbeTec ET system.. New Microbiol.

[82] Mitarai S, Tanoue S, Sugita C, Sugihara E, Tamura A (2001). Potential use of Amplicor PCR kit in diagnosing pulmonary tuberculosis from gastric aspirate.. J Microbiol Methods.

[83] Moore DF, Curry JI (1995). Detection and identification of Mycobacterium tuberculosis directly from sputum sediments by Amplicor PCR.. J Clin Microbiol.

[84] Moore DF, Curry JI (1998). Detection and identification of Mycobacterium tuberculosis directly from sputum sediments by ligase chain reaction.. J Clin Microbiol.

[85] Ni Riain U, Cormican M, Flynn J (1998). Transport of digested decontaminated sputum specimens to a central laboratory for testing for M. tuberculosis by Amplicor MTB test.. Ir J Med Sci.

[86] Oh EJ, Park YJ, Chang CL, Kim BK, Kim SM (2001). Improved detection and differentiation of mycobacteria with combination of Mycobacterium Growth Indicator Tube and Roche COBAS AMPLICOR System in conjunction with Duplex PCR.. J Microbiol Methods.

[87] O'Sullivan CE, Miller DR, Schneider PS, Roberts GD (2002). Evaluation of Gen-Probe amplified mycobacterium tuberculosis direct test by using respiratory and nonrespiratory specimens in a tertiary care center laboratory.. J Clin Microbiol.

[88] Pfyffer GE, Kissling P, Wirth R, Weber R (1994). Direct detection of Mycobacterium tuberculosis complex in respiratory specimens by a target-amplified test system.. J Clin Microbiol.

[89] Pfyffer GE, Kissling P, Jahn EM, Welscher HM, Salfinger M (1996). Diagnostic performance of amplified Mycobacterium tuberculosis direct test with cerebrospinal fluid, other nonrespiratory, and respiratory specimens.. J Clin Microbiol.

[90] Pfyffer GE, Funke-Kissling P, Rundler E, Weber R (1999). Performance characteristics of the BDProbeTec system for direct detection of Mycobacterium tuberculosis complex in respiratory specimens.. J Clin Microbiol.

[91] Piersimoni C, Callegaro A, Scarparo C, Penati V, Nista D (1998). Comparative evaluation of the new gen-probe Mycobacterium tuberculosis amplified direct test and the semiautomated abbott LCx Mycobacterium tuberculosis assay for direct detection of Mycobacterium tuberculosis complex in respiratory and extrapulmonary specimens.. J Clin Microbiol.

[92] Piersimoni C, Scarparo C, Piccoli P, Rigon A, Ruggiero G (2002). Performance assessment of two commercial amplification assays for direct detection of Mycobacterium tuberculosis complex from respiratory and extrapulmonary specimens.. J Clin Microbiol.

[93] Piersimoni C, Olivieri A, Benacchio L, Scarparo C (2006). Current perspectives on drug susceptibility testing of Mycobacterium tuberculosis complex: the automated nonradiometric systems.. J Clin Microbiol.

[94] Pounder JI, Aldous WK, Woods GL (2006). Comparison of real-time polymerase chain reaction using the Smart Cycler and the Gen-Probe amplified Mycobacterium tuberculosis direct test for detection of M. tuberculosis complex in clinical specimens.. Diagn Microbiol Infect Dis.

[95] Putova I, Havelkova M, Svandova E (1996). Application of the Gen-Probe amplified MTD test (Mycobacterium tuberculosis Direct Test) in the diagnostics of tuberculosis.. Cent Eur J Public Health.

[96] Rajalahti I, Vuorinen P, Nieminen MM, Miettinen A (1998). Detection of Mycobacterium tuberculosis complex in sputum specimens by the automated Roche Cobas Amplicor Mycobacterium Tuberculosis Test.. J Clin Microbiol.

[97] Rantakokko-Jalava K, Marjamaki M, Marttila H, Makela L, Valtonen V (2001). LCx Mycobacterium tuberculosis assay is valuable with respiratory specimens, but provides little help in the diagnosis of extrapulmonary tuberculosis.. Ann Med.

[98] Ribeiro FK, Dettoni Vdo V, Peres RL, Vinhas SA, Co TR (2004). Evaluation of a commercial test based on ligase chain reaction for direct detection of Mycobacterium tuberculosis in respiratory specimens.. Rev Soc Bras Med Trop.

[99] Rohner P, Jahn EI, Ninet B, Ionati C, Weber R (1998). Rapid diagnosis of pulmonary tuberculosis with the LCx Mycobacterium tuberculosis assay and comparison with conventional diagnostic techniques.. J Clin Microbiol.

[100] Ruiz-Serrano MJ, Albadalejo J, Martinez-Sanchez L, Bouza E (1998). LCx: a diagnostic alternative for the early detection of Mycobacterium tuberculosis complex.. Diagn Microbiol Infect Dis.

[101] Rusch-Gerdes S, Richter E (2004). Clinical evaluation of the semiautomated BDProbeTec ET System for the detection of Mycobacterium tuberculosis in respiratory and nonrespiratory specimens.. Diagn Microbiol Infect Dis.

[102] Salajka F, Mezensky L, Pokorny A (2000). Commercial polymerase chain reaction test (Amplicor set) in the diagnosis of smear-negative pulmonary tuberculosis from sputum and bronchoalveolar lavage.. Monaldi Arch Chest Dis.

[103] Scarparo C, Piccoli P, Rigon A, Ruggiero G, Scagnelli M (2000). Comparison of enhanced Mycobacterium tuberculosis amplified direct test with COBAS AMPLICOR Mycobacterium tuberculosis assay for direct detection of Mycobacterium tuberculosis complex in respiratory and extrapulmonary specimens.. J Clin Microbiol.

[104] Sheehan S, Schroder G, Garland J, Lancaster T (1997). Detection of Mycobacterium tuberculosis in Respiratory Specimens Using the New Abbot LCx MTB Assay.. Clin Lab.

[105] Shetty N, Shemko M, Holton J, Scott GM (2000). Is the detection of Mycobacterium tuberculosis DNA by ligase chain reaction worth the cost: experiences from an inner London teaching hospital.. J Clin Pathol.

[106] Shim TS, Chi HS, Lee SD, Koh Y, Kim WS (2002). Adequately washed bronchoscope does not induce false-positive amplification tests on bronchial aspirates in the diagnosis of pulmonary tuberculosis.. Chest.

[107] Smith MB, Bergmann JS, Harris SL, Woods GL (1997). Evaluation of the Roche AMPLICOR MTB assay for the detection of Mycobacterium tuberculosis in sputum specimens from prison inmates.. Diagn Microbiol Infect Dis.

[108] Smith MB, Bergmann JS, Onoroto M, Mathews G, Woods GL (1999). Evaluation of the enhanced amplified Mycobacterium tuberculosis direct test for direct detection of Mycobacterium tuberculosis complex in respiratory specimens.. Arch Pathol Lab Med.

[109] Soini H, Agha SA, El-Fiky A, Viljanen MK (1996). Comparison of amplicor and 32-kilodalton PCR for detection of Mycobacterium tuberculosis from sputum specimens.. J Clin Microbiol.

[110] Stauffer F, Mutschlechner R, Hasenberger P, Stadlbauer S, Schinko H (1995). Detection of Mycobacterium tuberculosis complex in clinical specimens by a commercial polymerase chain reaction kit.. Eur J Clin Microbiol Infect Dis.

[111] Takakura S, Tsuchiya S, Isawa Y, Yasukawa K, Hayashi T (2005). Rapid detection of Mycobacterium tuberculosis in respiratory samples by transcription-reverse transcription concerted reaction with an automated system.. J Clin Microbiol.

[112] Thomsen VO (1998). Diagnosis of pulmonary tuberculosis. Application of gen-probe amplified Mycobacterium tuberculosis direct test.. Apmis.

[113] Tonjum T, Klintz L, Bergan T, Baann J, Furuberg G (1996). Direct detection of Mycobacterium tuberculosis in respiratory samples from patients in Scandinavia by polymerase chain reaction.. Clin Microbiol Infect.

[114] Tortoli E, Lavinia F, Simonetti MT (1997). Evaluation of a commercial ligase chain reaction kit (Abbott LCx) for direct detection of Mycobacterium tuberculosis in pulmonary and extrapulmonary specimens.. J Clin Microbiol.

[115] Tortoli E, Tronci M, Tosi CP, Galli C, Lavinia F (1999). Multicenter evaluation of two commercial amplification kits (Amplicor, Roche and LCx, Abbott) for direct detection of Mycobacterium tuberculosis in pulmonary and extrapulmonary specimens.. Diagn Microbiol Infect Dis.

[116] Viinanen AH, Soini H, Marjamaki M, Liippo K, Viljanen MK (2000). Ligase chain reaction assay is clinically useful in the discrimination of smear-positive pulmonary tuberculosis from atypical mycobacterioses.. Ann Med.

[117] Viveiros M, Pinheiro S, Moreira P, Pacheco T, Brum L (1999). Evaluation of a commercial ligase chain reaction assay for the diagnosis of pulmonary and extra-pulmonary tuberculosis.. Int J Tuberc Lung Dis.

[118] Vuorinen P, Miettinen A, Vuento R, Hallstrom O (1995). Direct detection of Mycobacterium tuberculosis complex in respiratory specimens by Gen-Probe Amplified Mycobacterium Tuberculosis Direct Test and Roche Amplicor Mycobacterium Tuberculosis Test.. J Clin Microbiol.

[119] Wang SX, Tay L (1999). Evaluation of three nucleic acid amplification methods for direct detection of Mycobacterium tuberculosis complex in respiratory specimens.. J Clin Microbiol.

[120] Wang JY, Lee LN, Chou CS, Huang CY, Wang SK (2004). Performance assessment of a nested-PCR assay (the RAPID BAP-MTB) and the BD ProbeTec ET system for detection of Mycobacterium tuberculosis in clinical specimens.. J Clin Microbiol.

[121] Wang JY, Lee LN, Hsu HL, Hsueh PR, Luh KT (2006). Performance assessment of the DR. MTBC Screen assay and the BD ProbeTec ET system for direct detection of Mycobacterium tuberculosis in respiratory specimens.. J Clin Microbiol.

[122] Welch K, Brown G, Jonas V, Ferraro MJ (1995). Performance of the Gen-Probe amplified Mycobacterium tuberculosis direct test in a laboratory that infrequently isolates Mycobacterium tuberculosis.. Diagn Microbiol Infect Dis.

[123] Wobeser WL, Krajden M, Conly J, Simpson H, Yim B (1996). Evaluation of Roche Amplicor PCR assay for Mycobacterium tuberculosis.. J Clin Microbiol.

[124] Yee YC, Gough A, Kumarasinghe G, Lim TK (2002). The pattern of utilisation and accuracy of a commercial nucleic acid amplification test for the rapid diagnosis of Mycobacterium tuberculosis in routine clinical practice.. Singapore Med J.

[125] Zolnir-Dovc M, Poljak M, Seme K, Rus A, Avsic-Zupanc T (1995). Evaluation of two commercial amplification assays for detection of Mycobacterium tuberculosis complex in respiratory specimens.. Infection.

[126] Hadgu A, Dendukuri N, Hilden J (2005). Evaluation of nucleic acid amplification tests in the absence of a perfect gold-standard test: a review of the statistical and epidemiologic issues.. Epidemiology.

[127] Whiting P, Rutjes AW, Reitsma JB, Bossuyt PM, Kleijnen J (2003). The development of QUADAS: a tool for the quality assessment of studies of diagnostic accuracy included in systematic reviews.. BMC Med Res Methodol.

[128] Zamora J AV, Muriel A, Khan KS, Coomarasamy A (2006). Meta-DiSc: a software for meta-analysis of test accuracy data.. BMC Medical Research Methodology.

[129] Collaboration C (1996). Cochrane Methods Group on Systematic Review of Screening and Diagnostic Tests: Recommended Methods..

[130] Deeks JJ (2001). Systematic reviews in health care: Systematic reviews of evaluations of diagnostic and screening tests.. Bmj.

[131] Pai M, McCulloch M, Enanoria W, Colford JM (2004). Systematic reviews of diagnostic test evaluations: What's behind the scenes?. ACP J Club.

[132] Clarke MOA (2003). Cochrane reviewer's handbook 4.2.0.. The Cochrane Library, Issue 2:.

[133] Lau J, Ioannidis JP, Schmid CH (1997). Quantitative synthesis in systematic reviews.. Ann Intern Med.

[134] Glas AS, Lijmer JG, Prins MH, Bonsel GJ, Bossuyt PM (2003). The diagnostic odds ratio: a single indicator of test performance.. J Clin Epidemiol.

[135] Littenberg B, Moses LE (1993). Estimating diagnostic accuracy from multiple conflicting reports: a new meta-analytic method.. Med Decis Making.

[136] Irwig L, Macaskill P, Glasziou P, Fahey M (1995). Meta-analytic methods for diagnostic test accuracy.. J Clin Epidemiol.

[137] Lijmer JG, Bossuyt PM, Heisterkamp SH (2002). Exploring sources of heterogeneity in systematic reviews of diagnostic tests.. Stat Med.

[138] Rutjes AW, Reitsma JB, Di Nisio M, Smidt N, van Rijn JC (2006). Evidence of bias and variation in diagnostic accuracy studies.. Cmaj.

[139] Woods GL (2001). Molecular techniques in mycobacterial detection.. Arch Pathol Lab Med.

[140] Walter SD, Irwig L, Glasziou PP (1999). Meta-analysis of diagnostic tests with imperfect reference standards.. J Clin Epidemiol.

[141] (2000). Diagnostic Standards and Classification of Tuberculosis in Adults and Children. This official statement of the American Thoracic Society and the Centers for Disease Control and Prevention was adopted by the ATS Board of Directors, July 1999. This statement was endorsed by the Council of the Infectious Disease Society of America, September 1999.. Am J Respir Crit Care Med.

[142] Pai M, McCulloch M, Gorman JD, Pai N, Enanoria W (2004). Systematic reviews and meta-analyses: an illustrated, step-by-step guide.. Natl Med J India.

[143] Small PM, Perkins MD (2000). More rigour needed in trials of new diagnostic agents for tuberculosis.. Lancet.

[144] Walsh A, McNerney R (2004). Guidelines for establishing trials of new tests to diagnose tuberculosis in endemic countries.. Int J Tuberc Lung Dis.

[145] Lijmer JG, Mol BW, Heisterkamp S, Bonsel GJ, Prins MH (1999). Empirical evidence of design-related bias in studies of diagnostic tests.. Jama.

[146] (2001). CDC M tuberculosis nucleic acid amplification testing performance evaluation program. Centers for Disease Control and Prevention..

[147] Roos BR, van Cleeff MR, Githui WA, Kivihya-Ndugga L, Odhiambo JA (1998). Cost-effectiveness of the polymerase chain reaction versus smear examination for the diagnosis of tuberculosis in Kenya: a theoretical model.. Int J Tuberc Lung Dis.

[148] Perkins MD, Roscigno G, Zumla A (2006). Progress towards improved tuberculosis diagnostics for developing countries.. Lancet.

